# Fibulin-4 deficiency increases TGF-β signalling in aortic smooth muscle cells due to elevated TGF-β2 levels

**DOI:** 10.1038/srep16872

**Published:** 2015-11-26

**Authors:** N. W. M. Ramnath, L. J. A. C. Hawinkels, P. M. van Heijningen, L. te Riet, M. Paauwe, M. Vermeij, A. H. J. Danser, R. Kanaar, P. ten Dijke, J. Essers

**Affiliations:** 1 Department of Genetics, Cancer Genomics Centre Netherlands, Erasmus MC, Rotterdam, The Netherlands; 2 Department of Vascular Surgery, Erasmus MC, Rotterdam, The Netherlands; 3 Department of Molecular Cell Biology Leiden University Medical Centre, Leiden, The Netherlands, Cancer Genomics Centre; 4 Department of Gastroenterology-Hepatology, Leiden University Medical Centre, Leiden, The Netherlands; 5 Department of Pharmacology, Erasmus MC, Rotterdam, The Netherlands; 6 Department of Pathology, Erasmus MC, Rotterdam, The Netherlands; 7 Department of Radiation Oncology, Erasmus MC, Rotterdam, The Netherlands

## Abstract

Fibulins are extracellular matrix proteins associated with elastic fibres. Homozygous Fibulin-4 mutations lead to life-threatening abnormalities such as aortic aneurysms. Aortic aneurysms in Fibulin-4 mutant mice were associated with upregulation of TGF-β signalling. How Fibulin-4 deficiency leads to deregulation of the TGF-β pathway is largely unknown. Isolated aortic smooth muscle cells (SMCs) from Fibulin-4 deficient mice showed reduced growth, which could be reversed by treatment with TGF-β neutralizing antibodies. In Fibulin-4 deficient SMCs increased TGF-β signalling was detected using a transcriptional reporter assay and by increased SMAD2 phosphorylation. Next, we investigated if the increased activity was due to increased levels of the three TGF-β isoforms. These data revealed slightly increased TGF-β1 and markedly increased TGF-β2 levels. Significantly increased TGF-β2 levels were also detectable in plasma from homozygous *Fibulin-4*^*R/R*^ mice, not in wild type mice. TGF-β2 levels were reduced after losartan treatment, an angiotensin-II type-1 receptor blocker, known to prevent aortic aneurysm formation. In conclusion, we have shown increased TGF-β signalling in isolated SMCs from Fibulin-4 deficient mouse aortas, not only caused by increased levels of TGF-β1, but especially TGF-β2. These data provide new insights in the molecular interaction between Fibulin-4 and TGF-β pathway regulation in the pathogenesis of aortic aneurysms.

In developed countries 1–2% of all deaths are caused by aortic aneurysms and dissections^1^. In these countries the incidence of thoracic aortic aneurysms (TAA) is approximately 25 per 100,000 persons per year^2^. In general, a TAA is characterized by degeneration of the extracellular matrix (ECM) and vascular smooth muscle cells (SMCs), including (phenotypic) loss of SMCs and changes in SMC proliferation^3^,^4^,^5^. Several genes have been identified in both syndromic and non-syndromic forms of TAA, including ECM genes, genes encoding contractile proteins in SMCs and genes involved in the regulation of the transforming growth factor (TGF)-β pathway^6^,^7^,^8^.

There are three mammalian TGF-β isoforms: TGF-β1, -β2 and -β3. They are encoded by different genes, but show a high degree of amino acid sequence homology. All TGF-β isoforms bind to the latency-associated protein (LAP) and via the latent TGF-β binding protein (LTBP) to the ECM. Upon activation TGF-βs can bind to the type-II TGF-β receptors (TβRII), which recruit the type-I TGF-β receptor (TβRI), also called activin receptor-like kinase (ALK)-5. ALK-5 is transphosphorylated by TβRII and subsequently downstream SMAD proteins (i.e. SMAD2/3) are phosphorylated. Activated SMAD2 and -3 associate with SMAD4, leading to translocation to the nucleus where they interact with target gene promoters and regulate transcription of genes encoding for plasminogen activator inhibitor (PAI)-I, matrix metalloproteinases (MMPs) and ECM proteins^9^.

A crucial role for the TGF-β pathway in syndromes associated with TAA became evident from both studies in patients and in mouse models^10^,^11^,^12^,^13^,^14^. Although TAAs are usually associated with increased TGF-β signalling, this association has also been observed with loss of function mutations in TGF-β and TGF-β receptors^15^. The identification of these mutations has led to new insights in the pathogenesis of aneurysm formation, but the molecular mechanism remains to be elucidated.

Mutations in genes of the TGF-β pathway and the ECM lead to phenotypic and functional SMC loss: *Tgfbr2* mutations in Loeys-Dietz syndrome lead to decreased expression of SMC contractile proteins^5^. Furthermore, SMCs from mice with Marfan syndrome, another syndromic form of TAAs caused by mutations in the ECM glycoprotein Fibrillin-1, display an altered expression profile with morphological changes, but retain expression of vascular SMC markers^4^. In addition, increased TGF-β signalling inhibits proliferation of SMCs^16^.

Upregulated TGF-β signalling has been observed in another heritable form of TAA caused by a deficiency in the extracellular matrix protein Fibulin-4^13^,^17^,^18^,^19^,^20^. Fibulin-4 regulates proper elastogenesis by tethering lysyl oxidase to tropoelastin to facilitate crosslinking^21^,^22^. In Fibulin-4 deficient patients and mice elevated TGF-β signalling has been shown^12^,^13^,^20^. However, the exact mechanism by which Fibulin-4 deficiency leads to increased TGF-β signalling remains to be determined. To further investigate this we isolated SMCs from the aortic arch of hypomorphic Fibulin-4 (*Fibulin-4*^*R/R*^) mice, displaying a 4-fold reduction of Fibulin-4 expression. This leads to congenital vascular abnormalities in these mice, including TAAs and vascular tortuosity^12^. Heterozygous *Fibulin-4*^+/*R*^ mice, which have a 2-fold reduced Fibulin-4 expression, show minor irregularities and ECM changes in the aortic wall. Our data reveal that TGF-β signalling is enhanced in isolated SMCs derived from the aortas of Fibulin-4 deficient mice. We observed a decreased proliferation rate in *Fibulin-4*^*R/R*^ SMCs, which could be reverted by addition of TGF-β neutralizing antibodies. We found that this increased TGF-β signal transduction activity is not only associated with increased levels of TGF-β1, but especially with enhanced TGF-β2 levels. Increased levels of TGF-β2 could also be detected in blood and aortic tissue lysates of the *Fibulin-4*^*R/R*^ mice. Treatment of *Fibulin-4*^*R/R*^ mice with losartan, an angiotensin II type-1 receptor blocker, reduced the increased TGF-β2 levels in blood plasma. This study shows that increased TGF-β signalling in SMCs of Fibulin-4 deficient mice leads to decreased proliferation of SMCs and could be caused by increased bioavailability of TGF-β1 and especially TGF-β2.

## Results

### Characterization of SMCs derived from Fibulin-4 deficient aortas

**Figure 1:**
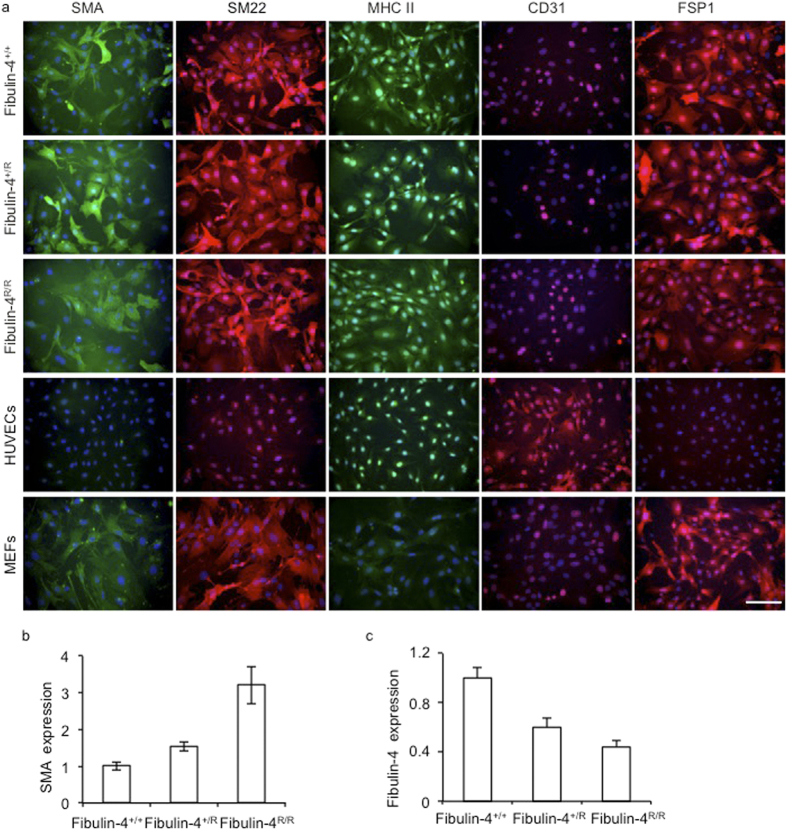
Characterization of isolated SMCs from the aortic arch. (**a**) Immunofluorescent staining of aortic SMCs isolated from *Fibulin-4*^+/+^, *Fibulin-4*^+/*R*^ and *Fibulin-4*^*R/R*^mice showed that these cells stained positively for SMA, SM22, MHC II and FSP1. The SMCs were negative for the endothelial marker CD31, while HUVECs were positive. HUVECs were negative for all other stainings. MEFs stained positive for SMA, SM22 and FSP1 and were negative for MHC II and CD31. Magnification 20x, scale bar 100 μm. (**b**) *Fibulin-4*^+/*R*^ and *Fibulin-4*^*R/R*^ SMCs show gradual increased SMA mRNA expression levels compared to *Fibulin-4*^+/+^ SMCs. (**c**) Fibulin-4 mRNA expression in SMCs. *Fibulin-4*^+/*R*^ and *Fibulin-4*^*R/R*^ SMCs show gradual decreased Fibulin-4 mRNA expression levels compared to *Fibulin-4*^+/+^ SMCs.

To examine TGF-β signalling in Fibulin-4 deficient SMCs, we isolated SMCs from the aortic arches of *Fibulin-4*^+/+^, *Fibulin-4*^+/*R*^ and *Fibulin-4*^*R/R*^ mice. To confirm that the cells we isolated were SMCs, the cells were analysed for the presence of SMC markers, including α-smooth muscle actin (α-SMA), smooth muscle specific protein-22 (SM22), smooth muscle myosin heavy chain II (MHC II) and fibroblast specific protein 1 (FSP1), which stains SMCs with a rhomboid phenotype^23^,^24^. Human umbilical vein endothelial cells (HUVECs) were taken along as positive control for CD31 staining and were negative for all other markers, while mouse embryonic fibroblasts (MEFs) were positive controls for FSP1, and SMA and SM22 staining^25^. Isolated SMCs showed positive staining for α-SMA, SM22, MHC II, FSP1 and were negative for CD31 (Fig. 1a) confirming the SMC phenotype. QPCR expression analysis also showed no detectable CD31 and von Willebrand Factor (an additional endothelial marker) mRNA expression (data not shown). α-SMA was highly expressed and seemed somewhat increased in *Fibulin-4*^+/*R*^ and *Fibulin-4*^*R/R*^ SMCs (Fig. 1b). Next, the levels of Fibulin-4 were analysed by QPCR. These data revealed that expression levels of Fibulin-4 mRNA in *Fibulin-4*^+/*R*^ and *Fibulin-4*^*R/R*^ SMCs were downregulated (Fig. 1c). These data show that we isolated a population of SMCs with a gradual reduced Fibulin-4 expression level, which we used for further cell biological and molecular analyses.

### TGF-β reduces proliferation of *Fibulin-4* ^*R/R*^ SMCs

**Figure 2:**
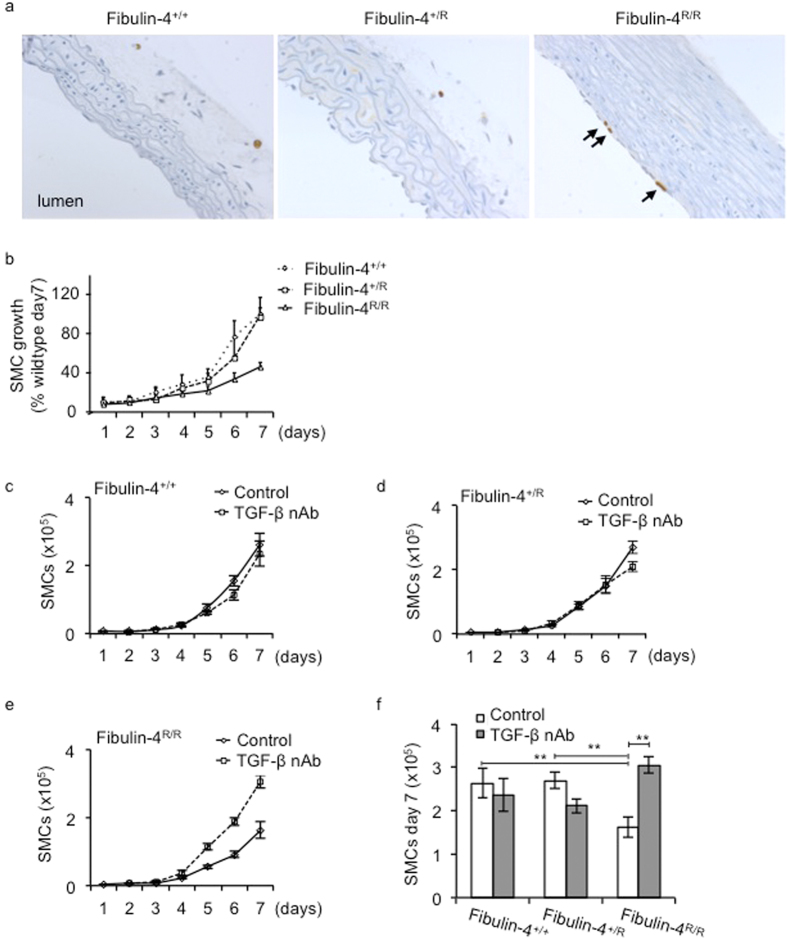
Reduced proliferation of *Fibulin-4*^*R/R*^ SMCs is reversed by inhibition of the TGF-β pathway. (**a**) Increased BrdU uptake indicating active proliferation of endothelial cells is present in the aortic wall from 100 days old *Fibulin-4*^*R/R*^ mice and is indicated by the arrows. (**b**) Growth analyses of *Fibulin-4*^+/+^, *Fibulin-4*^+/*R*^ and *Fibulin-4*^*R/R*^ SMCs revealed a reduced growth starting from day 5 for *Fibulin-4*^*R/R*^ SMCs as compared to *Fibulin-4*^+/+^ SMCs (3 dishes per experiment were counted and the average of 3 independent experiments is shown). Treatment of (**c**) *Fibulin-4*^+/+^, (**d**) *Fibulin-4*^+/*R*^ and (**e,f**) *Fibulin-4*^*R/R*^ SMCs with TGF-β neutralizing antibodies (nAb) significantly increased proliferation of *Fibulin-4*^*R/R*^ SMCs from day 5. (**f**) At day 7 the number of treated *Fibulin-4*^*R/R*^ SMCs was significantly higher and comparable to the amount of *Fibulin-4*^+/+^ SMCs (*p < 0.05, **p < 0.01). Data represent 3 independent experiments performed in triplicate.

Previously we showed in 10 days old *Fibulin-4*^*R/R*^ mice increased BrdU uptake indicating increased proliferation of SMCs, leading to changes in the tunica adventitia of the aorta^12^. However, in adult *Fibulin-4*^*R/R*^ mice (100 days old) increased proliferation was observed specifically in the endothelial layer (Fig. 2a). No proliferation of SMCs in the adventitia or media of the aortic wall was observed. Next, we analysed proliferation rates of the SMCs with reduced Fibulin-4 expression *in vitro*. Figure 2b shows similar growth rates of all three genotypes until day 5, after which proliferation was decreased in *Fibulin-4*^*R/R*^ SMCs. As TGF-β can inhibit cell proliferation, we determined whether the reduced growth of *Fibulin-4*^*R/R*^ SMCs is a consequence of increased TGF-β activity. Therefore, SMCs were treated with TGF-β neutralizing antibodies (nAb), which neutralize all three TGF-β isoforms^26^,^27^. Treatment with the TGF-β nAb reversed the growth inhibition observed in *Fibulin-4*^*R/R*^ SMCs compared to *Fibulin-4*^+/+^ SMCs (Fig. 2c–f). On day 7 the number of *Fibulin-4*^*R/R*^ SMCs was significantly increased after treatment with TGF-β nAb compared to non-treated *Fibulin-4*^*R/R*^ SMCs. Moreover, proliferation was similar to *Fibulin-4*^+/+^ SMCs. These data indicate that Fibulin-4 deficiency leads to increased TGF-β, which inhibits proliferation of SMCs.

### Fibulin-4 expression regulates TGF-β signalling in aortic SMCs

**Figure 3:**
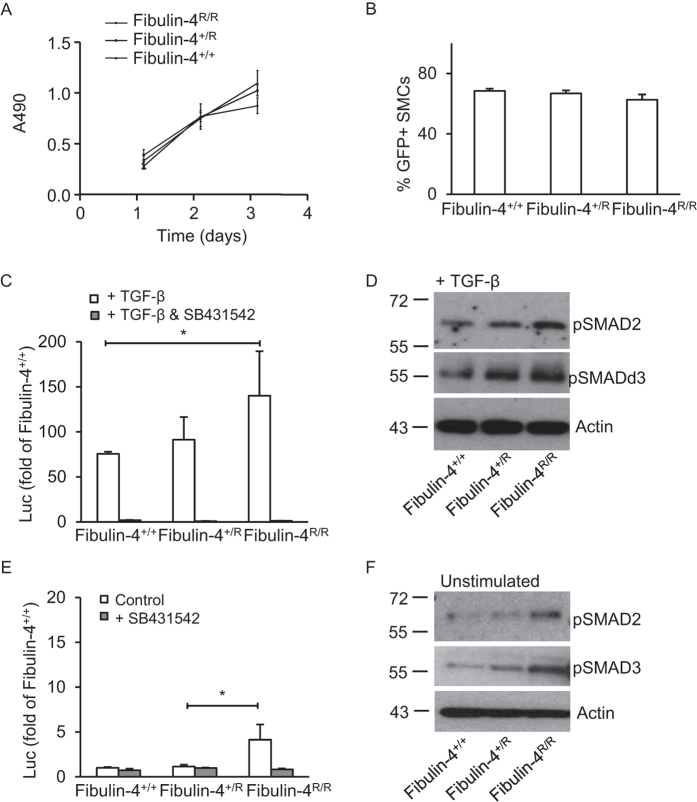
Increased TGF-β signalling in Fibulin-4 deficient SMCs. (**a**) *Fibulin-4*^+/+^, *Fibulin-4*^+/*R*^ and *Fibulin-4*^*R/R*^ SMCs show similar proliferation rates in the time course of the experiment (MTS proliferation assay) (**b**) Transfection with green fluorescent protein (GFP) encoding plasmids display no difference in percentage of transfected SMCs between *Fibulin-4*^+/+^, *Fibulin-4*^+/*R*^ and *Fibulin-4*^*R/R*^ SMCs. (**c**) The TGF-β response assay reveals a gradual increase in TGF-β activity in *Fibulin-4*^+/*R*^ and *Fibulin-4*^*R/R*^ SMCs compared to *Fibulin-4*^+/+^ SMCs, after stimulation with exogenous TGF-β. Data represent fold change relative to unstimulated *Fibulin-4*^+/+^ SMCs. Addition of the ALK-5 kinase inhibitor (SB431542) abolishes the TGF-β response in *Fibulin-4*^+/+^, *Fibulin-4*^+/*R*^ and *Fibulin-4*^*R/R*^ SMCs. (**d**) Western blot analyses for TGF-β signalling downstream mediators pSMAD2 and pSMAD3 on TGF-β stimulated SMCs show a gradual increase in TGF-β signalling in Fibulin-4 deficient SMCs compared to *Fibulin-4*^+/+^ SMCs. (**e**) Measurement of basal TGF-β activity (no stimulation with exogenous TGF-β) by the CAGA-luciferase reporter show increased TGF-β activity in *Fibulin-4*^*R/R*^ SMCs compared to *Fibulin-4*^+/+^ SMCs, which can be inhibited by the TβRI kinase inhibitor SB431542. (**f**) These data were confirmed by western blot analyses for pSMAD2 and pSMAD3. All data shown are representative for in total n = 3 independent experiments and all performed under serum starved conditions (*p < 0.05, **p < 0.01).

Since we observed that TGF-β neutralizing antibodies revert the decreased proliferation rates of *Fibulin-4*^*R/R*^ SMCs, we further analysed transcriptional consequences of increased TGF-β signalling in these cells using a SMAD3/SMAD4 dependent promoter transcriptional reporter construct (CAGA-luciferase)^28^. Although there was a difference in proliferation between different genotypes at later time points, this was not observed during the shorter duration of this assay (Fig. 3a). To determine whether transfection efficiency was similar between the different genotypes a green fluorescent protein (GFP) expressing construct was transfected and GFP expression determined. Flow cytometric analysis showed no differences between the percentages of GFP expressing *Fibulin-4*^+/+^, *Fibulin-4*^+/*R*^ and *Fibulin-4*^*R/R*^ SMCs (Fig. 3b) and thus no differences in transfection efficiencies among these different genotype. Next, we used the CAGA-luciferase reporter construct to assess TGF-β signalling activity in *Fibulin-4*^+/+^, *Fibulin-4*^+/*R*^ and *Fibulin-4*^*R/R*^ SMCs. Stimulation with TGF-β of *Fibulin-4*^+/+^, *Fibulin-4*^+/*R*^ and *Fibulin-4*^*R/R*^ SMCs showed a strong induction of luciferase activity, which was increased in a Fibulin-4 dose-dependent manner (Fig. 3c). Addition of the TβRI kinase inhibitor SB431542, a compound selectively blocking TGF-β type-I receptor kinase activity^29^, abolished TGF-β-induced transcriptional responses. Analysis of downstream pSMAD2 and pSMAD3 by western blotting revealed a gradual increase in SMAD2 and SMAD3 phosphorylation after stimulation with TGF-β in *Fibulin-4*^+/*R*^ and *Fibulin-4*^*R/R*^ SMCs compared to *Fibulin-4*^+/+^ SMCs (Fig. 3d), confirming the CAGA-luciferase reporter data. These data indicate that Fibullin-4 deficient cells show increased signalling upon exogenous TGF-β stimulation.

To explore whether basal TGF-β signalling is also affected in Fibulin-4 deficient cells, SMCs were transfected with the CAGA-luciferase reporter and TGF-β signalling without exogenous addition of TGF-β ligand was analysed. This showed that luciferase activity was already increased in untreated *Fibulin-4*^*R/R*^ SMCs compared to *Fibulin-4*^+/+^ SMCs (Fig. 3e). This could be reversed by SB431542, suggesting a TGF-β mediated effect. Western blot analysis showed gradually increased basal phosphorylation of SMAD2 and SMAD3 in untreated *Fibulin-4*^+/*R*^ and *Fibulin-4*^*R/R*^ SMCs (Fig. 3f). Taken together, these data indicate that increased phosphorylation of SMAD2/3 leads to enhanced transcriptional activation of downstream TGF- β signalling genes and reduced growth in Fibulin-4 mutant cells.

### Increased TGF-β1 and TGF-β2 levels in Fibulin-4 deficient SMCs

**Figure 4:**
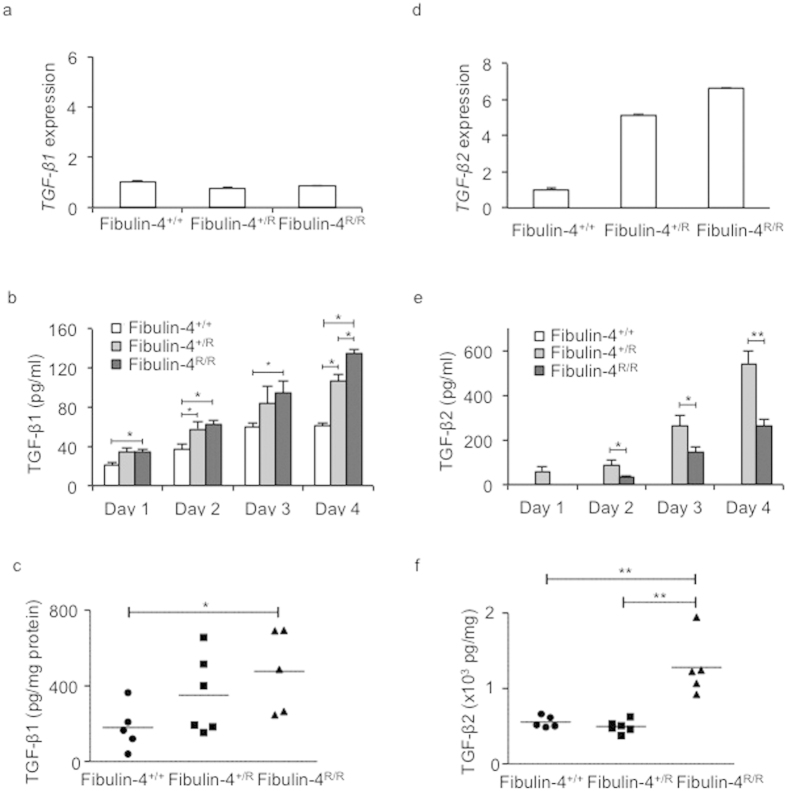
Strong increase of TGF-β2 levels in Fibulin-4 deficient SMCs. (**a**) *Fibulin-4*^+/*R*^ and *Fibulin-4*^*R/R*^ SMCs show equal *Tgf-β1* mRNA expression levels compared to *Fibulin-4*^+/+^ SMCs. (**b**) Increased TGF-β1 levels measured in conditioned medium (CM) from *Fibulin-4*^*R/R*^ SMCs compared to *Fibulin-4*^+/+^ CM on day 1–4 after serum starvation. *Fibulin-4*^+/*R*^ SMCs showed significant increased TGF-β1 levels on day 2 and 4 after serum starvation compared to *Fibulin-4*^+/+^ SMCs. Furthermore, on day 4 *Fibulin-4*^*R/R*^ SMCs show significant increased TGF-β1 levels compared to *Fibulin-4*^+/*R*^*SMCs* (n = 4 per day for each genotype). Two-way ANOVA analysis for genotype and between days p < 0.05. (**c**) Gradually increased TGF-β1 is also observed in aortic arch lysates of *Fibulin-4*^+/*R*^ (n = 6) and *Fibulin-4*^*R/R*^ mice (n = 5) compared to *Fibulin-4*^+/+^ aortas (n = 5). This increase is significant in *Fibulin-4*^*R/R*^ aortic arch lysates compared to *Fibulin-4*^+/+^ aortic arch lysates. (**d**) *Fibulin-4*^+/*R*^ and *Fibulin-4*^*R/R*^ SMCs show gradual increased *Tgf-β2* mRNA expression levels compared to *Fibulin-4*^+/+^ SMCs. (**e**) Measurement of TGF-β2 revealed markedly increased levels in CM of *Fibulin-4*^+/*R*^ and *Fibulin-4*^*R/R*^ SMCs, while TGF-β2 was undetectable in CM of *Fibulin-4*^+/+^ SMCs (experiments were performed in at least 3 independent experiments). Two-way ANOVA analysis for genotype and between days p < 0.05. (**f**) Measurements in aortic arch lysates display significantly increased TGF-β2 in *Fibulin-4*^*R/R*^ aortas compared to *Fibulin-4*^+/*R*^ and *Fibulin-4*^+/+^ aortas (*p < 0.05, **p < 0.01).

Since we observed increased basal TGF-β signalling in Fibulin-4 deficient SMCs, we analysed whether this was due to increased TGF-β levels. Subconfluent *Fibulin-4*^+/+^, *Fibulin-4*^+/*R*^ and *Fibulin-4*^*R/R*^ SMCs were serum-starved and conditioned medium was collected for 4 consecutive days to determine TGF-β1, − β2 and −β 3 levels. TGF-β3 levels were very low and did not differ between the different genotypes (data not shown). Although *Tgf-β1* mRNA levels in SMCs did not differ between the genotypes (Fig. 4a), TGF-β1 levels in *Fibulin-4*^*R/R*^ SMCs conditioned medium were higher compared to *Fibulin-4*^+/+^ SMCs (Fig. 4b). Conditioned medium from *Fibulin-4*^+/*R*^ SMCs showed intermediate TGF-β1 levels. To analyse whether the increased TGF-β1 levels were also observed *in vivo*, we prepared lysates from aortic arches of *Fibulin-4*^+/+^, *Fibulin-4*^+/*R*^ and *Fibulin-4*^*R/R*^ mice and measured TGF-β1 levels. These data revealed a similar gradual increase in TGF-β1 levels in aortic arch lysates of *Fibulin-4*^+/*R*^ and *Fibulin-4*^*R/R*^ mice (Fig. 4c).

**Figure 5:**
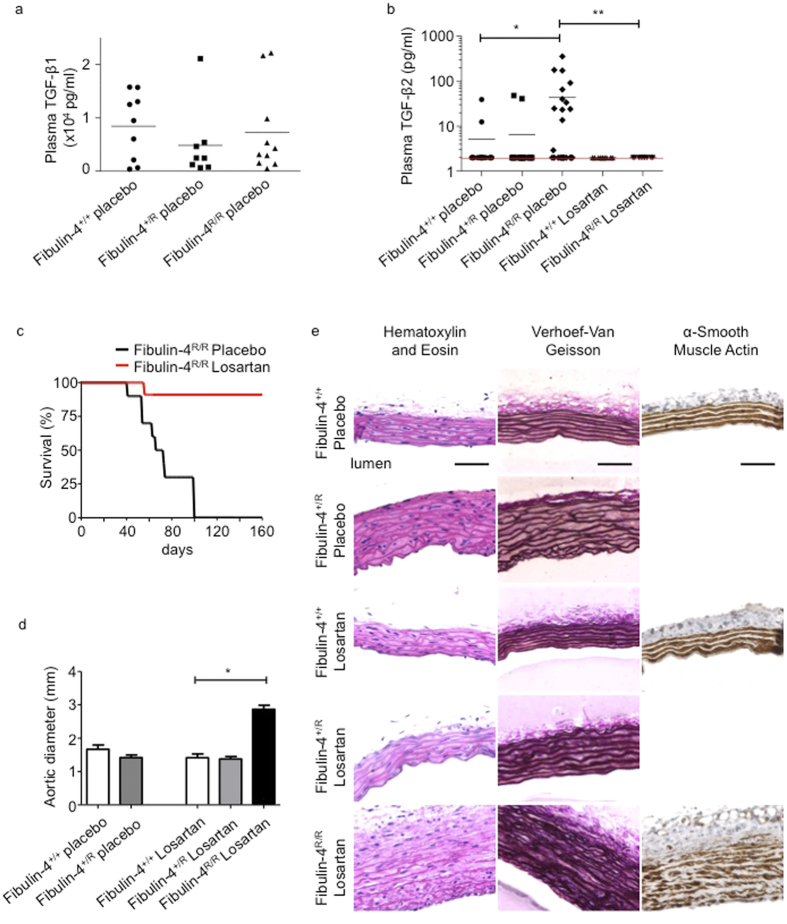
Increased levels of TGF-β2 are detectable in plasma from 100 days old *Fibulin-4*^*R/R*^ mice, which reduces on Losartan treatment. (**a**) TGF-β1 measurements in plasma samples showed no difference between placebo treated *Fibulin-4*^+/+^ (n = 9), *Fibulin-4*^+/*R*^ (n = 8) and *Fibulin-4*^*R/R*^ mice (n = 10). (**b**) TGF-β2 was detectable in plasma of 12 out of 24 placebo treated *Fibulin-4*^*R/R*^ mice compared to only 2 out of 15 in placebo treated *Fibulin-4*^+/+^ mice and 2 out of 19 in *Fibulin-4*^+/*R*^ mice. TGF-β2 levels are significantly higher in *Fibulin-4*^*R/R*^ mice compared to placebo treated *Fibulin-4*^+/+^ and *Fibulin-4*^+/*R*^ mice. Losartan treatment of *Fibulin-4*^*R/R*^ mice seemed to reduce the TGF-β2 levels (0 out of 9) as compared to placebo treated *Fibulin-4*^*R/R*^ mice. The red line indicates the detection of the ELISA (Chi-square p < 0.001). (**c**) Kaplan-Meier survival curve shows an increased survival of Losartan treated *Fibulin-4*^*R/R*^mice compared to placebo treated *Fibulin-4*^*R/R*^mice. (**d**) Aortic diameter of 160 days old placebo and losartan treated *Fibulin-4*^+/+^, *Fibulin-4*^+/*R*^ mice and Losartan treated *Fibulin-4*^*R/R*^ mice. Losartan treated *Fibulin-4*^*R/R*^mice have significantly enlarged aortic diameters compared to wild type mice, while there are no differences between placebo and Losartan *Fibulin-4*^+/*R*^mice and wild type mice. (**e**) HE, elastin and αSMA staining of ascending thoracic aortas. Placebo and losartan treated *Fibulin-4*^+/*R*^ mice (160 days old) show an increase in aortic wall thickness and some elastin breaks compared to *Fibulin-4*^+/+^ *mice*. Losartan treated *Fibulin-4*^*R/R*^mice show despite of their survival disrupted aortic wall architecture. In addition, despite losartan treatment there is loss of smooth muscle cell content in the media of *Fibulin-4*^*R/R*^ mice. (*p < 0.05, **p < 0.01).

Next, we analysed TGF-β2 expression in the SMCs. Fibulin-4^+/*R*^ and Fibulin-4^R/R^ SMCs showed >5-fold increased *Tgf-β2* mRNA expression levels (Fig. 4d). ELISA analysis on conditioned medium from *Fibulin-4*^+/+^, *Fibulin-4*^+/*R*^ and *Fibulin-4*^*R/R*^ SMCs revealed strongly increased TGF-β2 levels in medium from *Fibulin-4*^+/*R*^ and *Fibulin-4*^*R/R*^ SMCs compared to *Fibulin-4*^+/+^ SMCs, in which TGF-β2 was undetectable (Fig. 4e). TGF-β2 levels were already significantly higher in conditioned medium from *Fibulin-4*^+/*R*^ SMCs. Increased TGF-β2 levels were also detectable in *Fibulin-4*^*R/R*^ aortic arch lysates, when compared to aortic arch lysates from *Fibulin-4*^+/+^ and *Fibulin-4*^+/*R*^ mice (Fig. 4f). Given the increased TGF-β levels in SMCs and aortic tissue derived from Fibulin-4 deficient mice, we determined TGF-β1 and TGF-β2 levels in plasma samples from these mice. TGF-β1 levels were not significantly different among plasma from *Fibulin-4*^+/+^, *Fibulin-4*^+/*R*^ and *Fibulin-4*^*R/R*^ mice (Fig. 5a). In contrast, plasma TGF-β2 levels were very low in wild type mice and could be detected in 2 out of 15 *Fibulin-4*^+/+^ mice and 2 out of 19 *Fibulin-4*^+/*R*^ mice (Fig. 5b). TGF-β2 levels could be detected in plasma from 12 out of 24 *Fibulin-4*^*R/R*^ mice with significantly higher concentrations compared to *Fibulin-4*^+/+^ and *Fibulin-4*^+/*R*^ mice. These data show that specifically TGF-β2 levels in aortic tissue and plasma of Fibulin-4 deficient mice are strongly increased.

### Losartan treatment rescues lethality and lowers plasma TGF-β2 levels in *Fibulin-4* ^*R/R*^ mice

Next, adult mice were treated with Losartan, an angiotensin-II type-I receptor blocker, which prevents aortic root enlargement and reduces circulating TGF-β1 in a Marfan mouse model^30^. Compared to the increased secretion of TGF-β2 in placebo treated *Fibulin-4*^*R/R*^ mice we observed, TGF-β2 levels were not detectable in the 10 losartan treated *Fibulin-4*^*R/R*^ mice. Consistent with previous studies^31^, Losartan treatment of wild type, *Fibulin-4*^+/*R*^ and *Fibulin-4*^*R/R*^ mice showed improved survival rates of Losartan treated *Fibulin-4*^*R/R*^ mice until at least the age of 160 days compared to placebo treated *Fibulin-4*^*R/R*^ mice, which maximally survive until the age of 100 days (Fig. 5c). All losartan and placebo treated wild type and *Fibulin-4*^+/*R*^ mice survived at least until the duration of the experiment (data not shown). Despite improved survival, 160 days old Losartan treated *Fibulin-4*^*R/R*^ mice developed significantly enlarged aortic diameters compared to losartan treated wild type mice (Fig. 5d), and a thickened and degenerated aortic wall architecture as evidenced by fragmentation of its elastin layers (Fig. 5e). Previously, we showed a reduced SMA staining in the aortic wall of 100 days old Fibulin-4 deficient mice, indicative for SMC loss^31^. This SMC loss is not ameliorated by losartan treatment of *Fibulin-4*^*R/R*^ mice (Fig. 5e). Both placebo and Losartan treated *Fibulin-4*^+/*R*^ mice showed an increase in aortic wall thickness and minor elastin breaks compared to wild type mice, which was also previously observed in non-treated *Fibulin-4*^+/*R*^ mice^12^. These results show that lethality and increased plasma TGF-β2 levels in *Fibulin-4*^*R/R*^ mice can be reduced by losartan treatment, showing a causal relation between increased TGF**-**β signalling and lethality in aneurysmal Fibulin-4 mice.

## Discussion

In this study we show that TGF-β signalling is gradually enhanced in Fibulin-4 deficient SMCs in a Fibulin-4 dose-dependent manner and influences proliferation of these cells. The increased TGF-β signalling is consistent with increased TGF-β1 levels, and especially with increased TGF-β2 levels, detected in plasma from Fibulin-4 deficient mice.

Previous analyses on aortas from Fibulin-4 deficient mice showed increased TGF-β signalling associated with aneurysm formation by gene expression analysis and increased nuclear pSMAD2 staining in the SMCs of these aortas^12^. Isolation of aortic SMCs from these mice provided the opportunity to assess TGF-β signalling *in vitro*. *Fibulin-4*^*R/R*^ SMCs have a reduced proliferation rate compared to *Fibulin-4*^+/*R*^ and wild type SMCs, which is reversed by TGF-β inhibition. Reduced proliferation only takes place after a prolonged incubation time, which is most probably caused by the requirement of certain levels of TGF-β before it affects the proliferation rates of the SMCs. However, in the aortic wall local active TGF-β concentrations can be much higher, due to local activation of the ECM bound TGF-β. In our previous studies we found a hyperproliferation of SMCs as well as a decreased SMC content in the aortic wall of Fibulin-4 deficient mice^12^,^31^. The hyperproliferation of SMCs was specifically found in the adventitial layers of the aortic wall of newborns. Tsai *et al.* showed that TGF-β can transform from an inhibitor to a stimulant of SMC proliferation in the context of elevated SMAD3^32^. We observed a gradual increase in TGF-β signalling in Fibulin-4 deficient SMCs, which could be reverted by inhibition of TGF-β. These data indicate that the proliferation of Fibulin-4 deficient SMCs is reduced due to increased TGF-β signalling, thereby potentially contributing to aortic aneurysm formation.

ELISA analyses point to increased TGF-β1 levels in Fibulin-4 deficient SMCs and strongly increased TGF-β2 levels, also detected in plasma of *Fibulin-4*^*R/R*^ mice. The three TGF-β isoforms are involved in both overlapping and divergent roles. While *Tgf-β1* null mice develop an autoimmune-like inflammatory disease^33^ and *Tgf-β3* knockout mice show abnormal lung development and cleft palate^34^, *Tgf-β2* knockout mice have multiple developmental defects, including cardiovascular, pulmonary, skeletal, ocular, inner ear and urogenital manifestations^35^. *Tgf-β2* heterozygous mutations in patients result in a different phenotype compared to *Tgf-β2* knock-out mice^15^. TGF-β2 haplo-insufficiency predisposes for adult-onset vascular disease, including aortic tortuosity and dilation, cerebrovascular disease and mitral valve disease, which overlaps with the phenotype of Fibulin-4 deficient patients. The phenotype of the TGF-β2 deficient patients also shows overlap with other TGF-β signalopathies including Marfan syndrome, Loeys-Dietz syndrome, the aneurysm-osteoarthritis syndrome and similarly present with a paradoxical, probably compensatory, local increase in TGF-β1 and TGF-β2. Furthermore, increased *Tgf-β2* expression has been detected in patients with the Loeys-Dietz syndrome^36^. The fact that TGF-β2 haplo-insufficiency results in a cardiovascular phenotype and local increased TGF-β2, stresses the potential importance of TGF-β2 in the vasculopathy. For various TGF-β superfamily members it is known that their effects are very concentration dependent^37^,^38^. Very high or very low levels of these cytokines can have similar or opposite effects on cells. In addition, crucial in the regulation of TGF-β activity is its activation from the latent ECM-bound complexes. This might explain the, at first sight, contradictory findings. This also suggests that increased TGF-β2 expression is part of a common pathophysiologic process involved in aortic aneurysm formation in these syndromes. Whether it is a direct or indirect consequence of Fibulin-4 deficiency has to be determined in further studies.

We observed that specifically the TGF-β2 isoform is elevated and detected at higher levels in the conditioned medium from these cells and *in vivo* in aortic lysates and blood. TGF-β2 differs in its receptor binding properties from TGF-β1 and TGF-β3. While TGF-β1 and TGF-β3 have a high affinity for binding to TβRII, TGF-β2 primarily binds to the transforming growth factor type-III receptor (TβRIII), also called betaglycan, after which it presents the ligand to the TβRI-TβRII signalling complex^39^. Bee *et al.* showed a specific regulatory role for the TGF-β receptor-IIb (TβRIIb), an alternatively spliced variant of TβRII, in TGF-β2 signal transduction. TβRIIb mutations result in TGF-β2 dependent increased SMAD2 phosphorylation, which is involved in aortic aneurysm progression^40^. Human SMCs express TβRI, TβRII and TβRIII, while in SMCs derived from atherosclerotic lesions TβRII expression is decreased^41^. This indicates that alterations in TGF-β receptor expression probably contribute to the regulation of the TGF-β pathway. As our data point to markedly increased TGF-β2 levels in Fibulin-4 deficient SMCs, analyses on TGF-β receptors on these SMCs might further clarify the process of TGF-β regulation and determine its role in the pathogenesis of Fibulin-4 associated aortic aneurysms.

Increased TGF-β levels or TGF-β signalling is associated with multiple diseases. Enhanced TGF-β signalling is known to mediate a pathologic increase in ECM secretion and deposition and is causative for fibrosis in multiple disorders throughout the body^42^. Overexpression of TGF-β2 is likely to induce trabecular meshwork ECM deposition^43^ and increased ECM deposition is also observed in aortic aneurysm formation. TGF-β2 is also frequently overexpressed in malignant cancers, where it induces immunosuppression and stimulates metastasis formation^44^. TGF-β2 expression can be targeted with antisense oligonucleotides, which are currently under investigation in clinical trials^45^. As inhibition with pan TGF-β neutralizing antibodies is likely to induce side effects, aortic aneurysms associated with increased TGF-β2 might benefit from a TGF-β2 specific intervention decreasing systemic side effects by targeting the other isoforms. In Marfan patients, mouse models for Loeys-Dietz syndrome and transverse aortic constriction (TAC), losartan treatment prevents aortic aneurysm formation accompanied by reduced TGF-β1 levels in patients with Marfan syndrome, and reduced TGF-β1 and TGF-β2 levels in Loeys-Dietz syndrome and TAC mice^30^,^36^,^46^,^47^. Our data indicate that losartan could also serve as an important therapeutic agent. The exact mechanism how losartan treatment leads to reduced TGF-β signalling needs to be determined.

Fibulin-4 binds LTBP-1 with high affinity and therefore an important role for Fibulin-4 in the association of LTBP-1 with microfibrils is predicted. The large latent complex (LLC), which is formed by LTBP and LAP-bound TGF-β, is linked to microfibrils through binding of LTBP-1 to Fibrillin-1. Therefore, Fibulin-4 might be additionally involved in sequestering of the LLC through LTBP-1 binding^48^. We speculate that reduced Fibulin-4 levels lead to defective sequestering to the ECM and thereby increased free TGF-β1 and TGF-β2. In conclusion, these data show that SMC derived TGF-β2 is associated with aortic aneurysm formation and levels decrease upon losartan treatment, which improves survival of Fibulin-4 deficient mice. Specific intervention on TGF-β2 could provide more information on its role in the pathogenesis of aortic aneurysm formation. *In vitro* analyses on isolated SMCs provide the opportunity to determine the molecular link between Fibulin-4 and TGF-β pathway regulation, and to further unravel its role in aortic aneurysm formation.

## Material and Methods

### Animals

Mice containing the *Fibulin-4*^*R*^ allele were generated as previously described^12^. All mice used were bred in a C57BI/6J background and were kept in individually ventilated cages to keep animals consistently micro-flora and disease free. To avoid stress-related vascular injury, mice were earmarked and genotyped 4 weeks after birth. Animals were housed at the Animal Resource Centre (Erasmus University Medical Centre), which operates in compliance with the “Animal Welfare Act” of the Dutch government, using the “Guide for the Care and Use of Laboratory Animals” as its standard. As required by Dutch law, formal permission to generate and use genetically modified animals was obtained from the responsible local and national authorities. All animal studies were approved by an independent Animal Ethical Committee (Dutch equivalent of the IACUC).

### Treatment of mice

*Fibulin-4*^+/+^ and *Fibulin-4*^*R/R*^ mice received 0.6 gram/liter losartan (Sigma, Zwijndrecht, the Netherlands) or placebo in their drinking water as previously described^30^,^31^. Adult *Fibulin-4*^*R/R*^ mice and their wild type littermates were treated during 10 weeks or 18 weeks, starting at the age of 5 weeks. Blood samples from placebo or losartan treated *Fibulin-4*^+/+^ and *Fibulin-4*^*R/R*^ mice were obtained by cardiac puncture and collected in lithium heparin vials (Sarstedt, Numbrecht, Germany).

### Isolation of SMCs and cell culture

Vascular SMCs were isolated from the luminal side of the aortic arch from *Fibulin-4*^+/+^, *Fibulin-4*^+/*R*^ and *Fibulin-4*^*R/R*^ male mice. The tissue was washed with phosphate-buffered saline (PBS), cut into 5-mm pieces with the luminal side on 0.1% gelatine coated cell culture dishes and incubated. After 7–10 days, smooth muscle-like cell outgrowth was observed. SMCs were maintained in DMEM (Lonza, Leusden, the Netherlands), supplemented with 10% etal calf serum (HyClone, Thermo Scientific, Breda, the Netherlands), 100 U/ml penicillin and 100 μg/ml streptomycin (Sigma-Aldrich, Zwijndrecht, the Netherlands). Cells were used at passage 5–11.

### Immuno-fluorescent and –histochemical stainings

Subconfluent SMCs, Human Umbilical Vein Endothelial Cells (HUVECs), isolated as described before^49^, and Mouse Embryonic Fibroblasts (MEFs), isolated from 8 day old C57/Bl6 mouse embryos, were grown on coverslips and fixed in 1% paraformaldehyde. Cells were permeabilised with 0.1% Triton/PBS and blocked with PBS containing 1.5% bovine serum albumin/0.15% glycine (Sigma). Next, coverslips were incubated overnight at 4 °C with the primary antibodies; mouse anti-smooth muscle actin 1:1500 (Progen, Heidelberg, Germany), rabbit polyclonal anti-SM22 alpha antibody 1:400 (Abcam, Cambridge, UK), mouse monoclonal anti-smooth muscle myosin heavy chain II 1G12 1:500 (Abcam, Cambridge, UK), rabbit anti-CD31 1:800 (Santa Cruz Biotechnologies, Santa Cruz, USA) and rabbit anti-Fibroblast Specific Protein1 (FSP-1)/S100A4 1:1600 (Millipore, MA, USA). The next day cells were incubated with secondary antibodies anti-mouse alexafluor 488 1:1000 (Molecular Probes, Eugene, Oregon) for SMA and MHC II and anti-rabbit alexafluor 594 1:1000 (Molecular Probes, Eugene, Oregon) for SM22, CD31 and FSP-1, and mounted with DAPI. Slides were analysed with the LEICA DMRBE Aristoplan Microscope equipped with the Hamamatsu ORCA-ER Camera. Pictures were taken at 25x magnification. To analyse *in vivo* SMC content and proliferation 4 μm sections of paraffin embedded aortas were stained with haematoxylin and eosin, elastin (Verhoeff-van Gieson), and α-smooth muscle actin as described before^31^. BrdU staining was performed according to the manufacturers’ protocol (Roche, Basel, Switzerland).

### Proliferation assay

Fibulin-4^+/+^, Fibulin-4^+/*R*^ and Fibulin-4^R/R^ SMCs were seeded in triplicate in 6 cm dishes (5000 cells/well) and allowed to attach overnight. Next, cells were treated with TGF-β neutralizing antibodies (kindly provided by Dr. E. de Heer, Leiden University Medical Centre, Dept. of Pathology^26^,^27^) and counted every day using a Burker cell counting chamber. Medium was replaced every other day. The MTS proliferation assay was performed according to the manufacturer’s instructions (Promega, Madison, USA). In short, SMCs were seeded in 96-well plates (1500 cells/well) and allowed to attach overnight. At day-1, -2 and -3 medium was changed to 100 μl complete DMEM + 20 μl MTS substrate and the metabolic activity of the cells was analysed by absorbance change at 490 nm after 2 hours.

### TGF-β response assay

TGF-β response in SMCs was determined using (CAGA)_12_−MLP−Luciferase promoter reporter construct^28^. This construct contains 12 palindromic repeats of the SMAD3/4 binding element derived from the *PAI-1* promoter and was shown to be highly specific and sensitive to TGF-β. The assay was performed as described previously^29^. In short SMCs were seeded in 1% gelatin coated 24-well plates and allowed to attach overnight. Subconfluent cells were transfected using Lipofectamin 2000 (Invitrogen, Carlsbad, California, USA) according to the manufacturer’s protocol. A β-galactosidase plasmid was co-transfected to correct for transfection efficiency. After 6 hours, medium was changed to DMEM containing 10% FCS and the cells were incubated for 24 hours. Next, cells were serum-starved overnight and stimulated with 5 ng/ml TGF-β3 (kindly provided by Kenneth K. Iwata, OSI, Inc., New York, USA) in the presence or absence of 10 uM SB431542 (Tocris/R&D systems, Abington, UK) for 6 hours. After stimulation the cells were washed, lysed and luciferase activity was determined according to the manufacturer’s protocol (Promega). β-Galactosidase activity in the lysates was determined using β-gal substrate (0.2 M H_2_PO_4_, 2 mM MgCl_2_, 4 mM ortho-nitrophenyl-phosphate, 0.25% β-mercaptoethanol) and measuring absorbance change at 405 nm. The luciferase count was corrected for β-galactosidase activity. The relative increase in luciferase activity was calculated versus controls. All experiments were performed at least three times in triplicate. To determine the transfection efficiency of *Fibulin-4*^+/+^, *Fibulin-4*^+/*R*^ and *Fibulin-4*^*R/R*^ SMCs they were transfected with a GFP plasmid as described above, trypsinised and fixed with 1% PFA. Subsequently, SMCs were analysed with flow cytometry for the percentage of GFP transfected SMCs compared to the total amount of SMCs.

### Western blot analysis

Western blot analysis was performed as described before^50^. In short, equal amounts of protein (DC protein assay, Bio-Rad Laboratories, Hercules, CA, USA) were separated on 10% SDS−polyacrylamide gel electrophoresis under reducing conditions. Proteins were transferred to nitrocellulose membranes (Whattman, Dassel, Germany) and blocked with 5% milk powder in Tris-HCl buffered saline containing 0.05% Tween-20 (Merck, Darmstadt, Germany). After washing, blots were overnight incubated with rabbit anti-pSMAD2 (Cell signaling Technologies, USA) and rabbit anti-pSMAD3 (kindly provided by Dr. E. Leof, Mayo Clinic, Rochester, MN, USA) followed by horseradish peroxidase-conjugated secondary antibodies (all GE Healthcare, Waukesha, WI, USA). Detection was performed by chemoluminescence according to the manufacturer’s protocol (Pierce, Rockford, IL, USA). Afterwards, blots were stripped and reprobed with mouse anti-β-actin antibodies as a loading control.

### RNA isolation and real-time PCR

RNA was isolated using RNeasy Mini Kit according to the manufacturer’s instructions (Qiagen, Hilden, Germany). RNA concentration and purity was determined spectrometrically. Complementary DNA synthesis was performed using random primers. cDNA samples were subjected to 40 cycles real-time PCR analysis using maxima SYBR Green qPCR Master Mix 2× (Fermentas, Vilnius, Lithuania) and primers shown in Table 1. Reactions were performed in triplicates for each sample. Product specificity was determined by melting curve analysis and gel electrophoresis. The average Ct values of the triple reactions were calculated for each gene and all values were normalized for cDNA content by *Hprt* expression. The levels of fold-change for each gene were calculated relative to the gene expression levels in baseline wild type SMCs. RNA isolated from HUVECs and fibroblasts were used as controls for the genes analysed.

### TGF-β ELISAs

SMC conditioned medium was prepared by seeding the cells and growing them to subconfluence. Medium was changed to serum-free DMEM, containing antibiotics as described above, and incubated for 4 days. Samples were collected every day and frozen at −20 until analysis. Lysates were prepared from aortic arches of 14–15 week old *Fibulin-4*^+/+^, *Fibulin-4*^+/*R*^ and *Fibulin-4*^*R/R*^ mice and protein amounts were determined (Pierce BCA protein assay kit, Thermo Scientific). Total TGF-β1, TGF-β2, and TGF-β3 levels in CM samples, aortic arch lysates and plasma samples were determined by commercially available duo-sets (R&D Systems) using transient acidification as described before^51^.

### Statistical analysis

Data are presented as mean ± SEM. The non-parametric Mann-Whitney U-test and unpaired student’s t-test were performed to analyse the specific sample groups for significant differences. The two-way ANOVA test was used to test significant differences between independent variables. A p-value of <0.05 was considered to indicate a significant difference between groups. All analyses were performed using IBM SPSS Statistics version 20.0 and 22.0 (SPSS Inc., Chicago, IL, USA).

## Additional Information

**How to cite this article**: Ramnath, N. W. M. *et al.* Fibulin-4 deficiency increases TGF-β signalling in aortic smooth muscle cells due to elevated TGF-β2 levels. *Sci. Rep.* **5**, 16872; doi: 10.1038/srep16872 (2015).

## Figures and Tables

**Table 1:** Primers used for quantitative real time PCR.

Genes	Forward primers	Reverse primers
Fibulin-4	5′-GGGTTATTTGTGTCTGCCTCG-3′	5′-TGGTAGGAGCCAGGAAGGTT-3′
SMA	5′-GTCCCAGACATCAGGGAGTAA-3′	5′-TCGGATACTTCAGCGTCAGGA-3
TGF-β1	5′-CAACAATTCCTGGCGTTACC-3′	5′-TGCTGTCACAAGAGCAGTGA-3′
TGF-β2	5′-CCGCCCACTTTCTACAGACCC-3′	5′-GCGCTGGGTGGGAGATGTTAA-3′

Forward and reverse primers are displayed for each gene from 5′ to 3′.
